# Supplementary material for: A Custom Panel for Profiling Microglia Gene Expression
Source: Cells. 2024 Apr 4;13(7):630. doi: 10.3390/cells13070630 (PMC11012202; doi:10.3390/cells13070630)
Supplement: Supplementary file 1 [file cells-13-00630-s001.zip › Supplementrary file 1_File containing gene names from Venn diagrams and Elbow plot for K-means clustering.pdf]

A Custom Panel for Profiling Microglia Gene Expression

Phani Sankar Potru [1], Natascha Vidovic [1], Susanne Wiemann [1], Tamara Ruß [1], Marcel Trautamann [2], and Björn Spittau [1,]

Supplementary data:

(a)

Names	total	elements
Adult MG vs BV2_Up AdultMG vs BMMn_Up AdultMG vs pMG_Up	62	Nlrp3 P2Ry13 Ctss Ccr8 Nos2 C3Ar1 Siglec-H Casp4 Il13 Fcrls Cd9 Cd33 Il3 Cass4 Tyrobp Il27 P2Rx7 Tgfbr2 Bmp2 Il6 Cxcl2 Il1B Bin1 Csf1R Gpr34 Tgfa Ccr5 Bmp3 Casp1 Cx3Cr1 Picalm Nrtn Ntf3 Tmem119 Il10Rb Bmp5 Il1A Il10Ra Sall1 Cst3 Tnf Tlr2 Hexb P2Ry12 Ccl4 Tnfsf13B Abi3 B2M Ccr4 Tgfbr1 Ccr6 Grn Pycad C1Qb Slc2A5 Clec7A Trem2 Ctsd Olfml3 Bmp10 Cxcl9 Il21
AdultMG vs BMMn_Up AdultMG vs pMG_Up	8	Tnfsf11 Il9 Cst7 Ccl9 Il17A Cd68 Lpl Ccl3
Adult MG vs BV2_Up AdultMG vs BMMn_Up	34	Tgfb2 Bmp4 Aif1 Il17F Cd14 Nlrp5 Itgav Lamp2 Ccnd1 Timp2 Lrp1 Ms4A7 Il7 Axl Bmp7 Tnfrsf11B Bmp8B Nodal Lrp6 Il18 Sparc Sppl2A Mrc1 Ngf Lamp1 Apbb3 Cxcl10 Apoe Ldhb Il5 Tnfsf10 Gdnf Bmp1 Fgf2
Adult MG vs BV2_Up AdultMG vs pMG_Up	17	Ccl6 Cd74 Tgfb1 Ccr3 C5Ar1 Apoc2 Ptk2B Cd22 Rin3 Ms4A4C Ccr2 Sorl1 Il12A Il16 Il15 Plcg2 Nlrc4
AdultMG vs BMMn_Up	9	Ccl2 Rabep1 Gpnmb Ctsl Ccl7 Spp1 Ctsb Igf1 Csf1
AdultMG vs pMG_Up	3	Slc16A3 Itgax Cd72
Adult MG vs BV2_Up	17	Zyx Stat2 Jak1 Pdgfc Tgfbr3 Tmsb4X Cxcr4 Bmp6 Stat1 Tgfb3 Bdnf Cxcl5 Il1R1 Apoc1 Thbs1 Apobec1 C3

(b)

Names	total	elements
AdultMG vs BMMn_Down AdultMG vs pMG_Down	3	Lyz2 Thbs1 C3
Adult MG vs BV2_Down AdultMG vs BMMn_Down	1	Tfec
Adult MG vs BV2_Down AdultMG vs pMG_Down	5	Fabp5 Igf1 Spp1 Gpnmb Mfge8
AdultMG vs BMMn_Down	7	Cxcr4 Zyx Sorl1 Cybb Il12A Pcna Cd72
AdultMG vs pMG_Down	17	Ngf Bmp6 Tgfb3 Tgfbr3 Bmp7 Tnfrsf11B Fth1 Tgfb2 Msr1 Vegfa Timp2 Bdnf Lrp6 Bmp4 Ms4A7 Il1R1 Pdgfc
Adult MG vs BV2_Down	7	Nlrc3 Lpl Ccl3 Mif Lilrb4 Cd36 Lgals3

(C)

Names	total	elements
BV2 vs BMMn_Up pMG vs BMMn_Up pMG vs BV2_Up	6	Sall1 Fth1 C3Ar1 Ccl7 Ctsb Ctsd
pMG vs BMMn_Up pMG vs BV2_Up	48	Bmp6 Tgfb3 Bmp7 Tnfrsf11B Tgfb2 Msr1 Cst3 Tlr2 Hexb Bdnf Lrp6 Bmp4 Il18 Aif1 Sparc Sppl2A Ccl5 Fcrls Il1R1 Pdgfc Ngf Lamp1 Cxcl10 Tgfbr2 Apoe Tgfbr3 Ldhb Itgav Lamp2 Cxcl2 Ccnd1 Timp2 Tnfsf10 C1Qb Lrp1 Gpr34 Tgfa Ms4A7 Il7 Gdnf Bmp1 Bmp3 Cx3Cr1 Axl Olfml3 Tmem119 Bmp5 Fgf2
BV2 vs BMMn_Up pMG vs BMMn_Up	17	Fabp5 Ctsl Lilrb4 Ccl4 Spp1 Cd14 Ccl3 Ccl2 Il17A Igf1 Cd68 Slc2A5 Csf1 Itga5 Trem2 Mfge8 Gpnmb
pMG vs BV2_Up	41	P2Ry13 Cxcr4 Il10Ra Stat1 Bmp8B Gnas P2Ry12 Nodal Siglec-H Zyx Sorl1 Cxcl5 Il17F Il12A Ccr3 C5Ar1 Cd33 Stat2 Il3 Jak1 Mrc1 Ccr4 Il27 Apbb3 Tgfbr1 Lyz2 Bmp2 Nlrp5 Apoc1 Il5 Nlrc4 Thbs1 Pycad Tmsb4X Apobec1 Picalm Bmp10 Ntf3 C3 Cxcl9 Il21
pMG vs BMMn_Up	2	Il6 Grn
BV2 vs BMMn_Up	13	Nlrc3 Lpl Tnf Mif Il9 Il13 Cd9 Slc16A3 P2Rx7 Ccl9 Rabep1 Cd36 Lgals3

(d)

Names	total	elements
BV2 vs BMMn_Down pMG vs BMMn_Down pMG vs BV2_Down	3	Cd74 Plcg2 Cd72
pMG vs BMMn_Down pMG vs BV2_Down	6	Tfec Cass4 Slc16A3 Panx1 Clec7A Casp1
BV2 vs BMMn_Down pMG vs BMMn_Down	23	Cxcr4 Cd22 Il10Ra Rin3 Ccl6 Tgfb1 Ms4A4C Ccr2 Siglec-H Sorl1 Cybb Il12A Ccr3 Cd33 Il16 Tnfsf13B Pcna Abi3 Apoc2 Il1B Ptk2B C3 Cxcl9
pMG vs BV2_Down	18	Nlrp3 Nlrc3 Lpl Mif Il9 Lilrb4 Ccl4 Itgax Spp1 P2Rx7 Cst7 Ccl9 Ccl3 Cd68 Cd36 Mfge8 Gpnmb Lgals3
BV2 vs BMMn_Down	38	P2Ry13 Bmp6 Stat1 Tnfrsf11B Bdnf P2Ry12 Zyx Sparc Cxcl5 C5Ar1 Jak1 Mrc1 Il15 Il1R1 Pdgfc Ngf Il27 Tgfbr1 Lyz2 Tgfbr2 Bmp2 Tgfbr3 Ldhb Apoc1 Nlrc4 Pycad Thbs1 C1Qb Tmsb4X Gpr34 Apobec1 Cx3Cr1 Picalm Axl Bmp10 Olfml3 Tmem119 Il21

Figure S1: Tables of the with the gene names and the groups compared corresponding to the Venn diagrams.

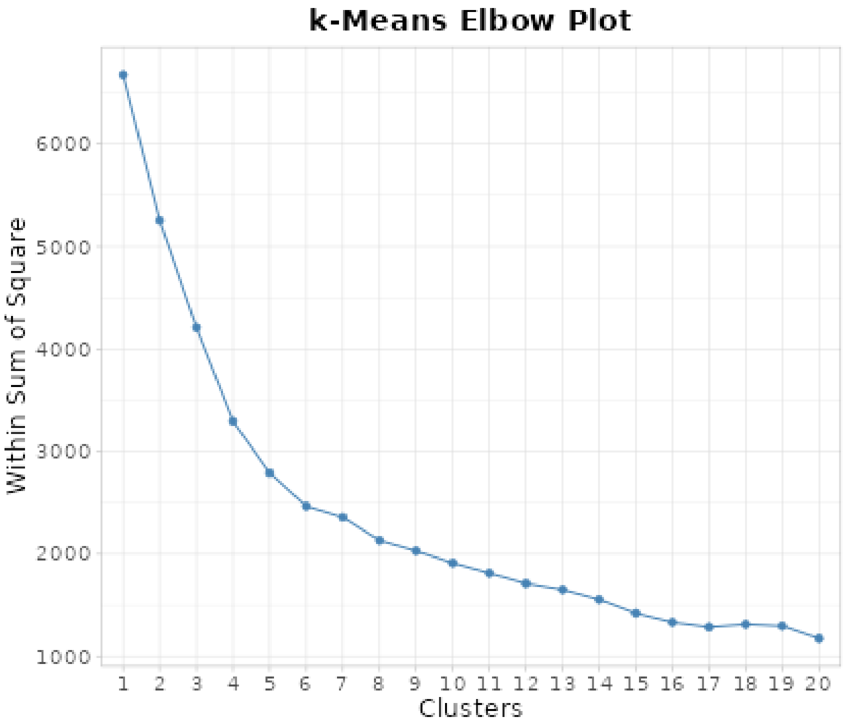

Figure S2:Elbow plot used for determining the number of k-means clusters.
